# Handling of thermal paper: Implications for dermal exposure to bisphenol A and its alternatives

**DOI:** 10.1371/journal.pone.0178449

**Published:** 2017-06-01

**Authors:** Meghan R. Bernier, Laura N. Vandenberg

**Affiliations:** Department of Environmental Health Sciences, School of Public Health and Health Sciences, University of Massachusetts – Amherst, Amherst, Massachusetts United States of America; Universidad Miguel Hernandez de Elche, SPAIN

## Abstract

Bisphenol A (BPA) is an endocrine disrupting chemical used in a wide range of consumer products including photoactive dyes used in thermal paper. Recent studies have shown that dermal absorption of BPA can occur when handling these papers. Yet, regulatory agencies have largely dismissed thermal paper as a major source of BPA exposure. Exposure estimates provided by agencies such as the European Food Safety Authority (EFSA) are based on assumptions about how humans interact with this material, stating that ‘typical’ exposures for adults involve only one handling per day for short periods of time (<1 minute), with limited exposure surfaces (three fingertips). The objective of this study was to determine how individuals handle thermal paper in one common setting: a cafeteria providing short-order meals. We observed thermal paper handling in a college-aged population (n = 698 subjects) at the University of Massachusetts’ dining facility. We find that in this setting, individuals handle receipts for an average of 11.5 min, that >30% of individuals hold thermal paper with more than three fingertips, and >60% allow the paper to touch their palm. Only 11% of the participants we observed were consistent with the EFSA model for time of contact and dermal surface area. Mathematical modeling based on handling times we measured and previously published transfer coefficients, concentrations of BPA in paper, and absorption factors indicate the most conservative estimated intake from handling thermal paper in this population is 51.1 ng/kg/day, similar to EFSA’s estimates of 59 ng/kg/day from dermal exposures. Less conservative estimates, using published data on concentrations in thermal paper and transfer rates to skin, indicate that exposures are likely significantly higher. Based on our observational data, we propose that the current models for estimating dermal BPA exposures are not consistent with normal human behavior and should be reevaluated.

## Introduction

Bisphenol A (BPA) is an endocrine disrupting chemical that has been widely studied in both controlled laboratory experiments and human populations [1, 2]. More than 100 epidemiology studies suggest associations between BPA exposures and an increased risk of adverse health outcomes including cardiovascular disease, obesity, diabetes, ADHD, male sexual dysfunction and others [3]. Evaluations of biomonitoring studies have revealed extensive, ubiquitous exposures to this compound in human populations from around the world [4–8]. Furthermore, hundreds of laboratory animal studies suggest that low doses of BPA can affect endocrine-sensitive endpoints and developing tissues including the nervous system, both male and female reproductive tissues, the immune system, mammary gland, and other metabolic tissues [9–12]. These studies also suggest that there is increased sensitivity to BPA and other endocrine disruptors during vulnerable periods of development including gestation and the perinatal period [13, 14].

BPA is a high production volume chemical that is used in a range of consumer products including plastics, reusable food and beverage containers, food can linings, medical equipment, and other consumer goods [15–17]. BPA leaches from these products at low concentrations, even when used according to the manufacturer’s instructions. Based on the detection of BPA in food and food packaging, it has been assumed by regulatory agencies that oral exposures are the major source of human exposures [18, 19]. This led some agencies to assert that only animal studies that use oral routes of BPA administration are relevant to human exposures and therefore only these studies should be used by risk assessors to evaluate the potential health effects of BPA exposures.

More recent evaluations have revealed the presence of BPA in products that come in contact with skin including cosmetics and thermal paper [20, 21]. In many thermal papers used as cash register receipts and tickets for airlines and trains, BPA is applied to the printing surface to be used as a heat-activated developer [22]. In these papers, the coating includes milligrams of unbound BPA per gram of paper [21–25]. When a laser is directed on the paper, the heat causes BPA to react with the thermal paper dye, producing a color-developing complex. Whereas BPA used in plastics and can linings are found largely in a polymerized form like polycarbonate or PVC, BPA applied to thermal paper is free, thus relatively large exposures from these materials may occur after normal handling [17, 26]. Studies evaluating urinary concentrations of BPA and its metabolites suggest slightly elevated exposures in individuals that work as cashiers [27]. Work simulations involving handling of thermal paper receipts also suggest that typical occupational exposures can increase urinary concentrations of BPA and its metabolites approximately 3-times (from 1.8 ng/ml prior to handling to 5.8 ng/ml after handling [28]).

Understanding sources and routes of exposure to BPA and other endocrine disrupting chemicals is essential to evaluate toxicokinetic parameters, including the amount of time that the compound can circulate in the body in the unconjugated form before it is metabolized [29–31]. When BPA enters the body via the oral route, it is absorbed into the mesenteric blood vessels, transported to the liver, and rapidly metabolized in a process referred to as ‘first pass metabolism’ [31]. Such processes mean that the majority of BPA that circulates in the bloodstream following oral exposure is in the conjugated form (e.g., BPA-glucuronide, BPA-sulfate) although some unconjugated BPA does reach circulation [29, 32, 33]. In contrast, when BPA enters the body via alternative routes (e.g., dermal or inhalation), it circumvents first-pass metabolism, allowing significantly more unconjugated BPA to circulate in the bloodstream [29–31]. These toxicokinetic data suggest that the route of exposure can have a large influence on the concentration of BPA that circulates as unconjugated BPA [19, 34]. This is important because, for BPA, only the unconjugated form can bind to estrogen receptor, leading some groups to conclude that only the unconjugated form is biologically active and therefore hazardous [35, 36]. In rodents, route of exposure affects the maximal blood concentrations and other toxicokinetic parameters, even though the biological effect observed may be the same regardless of exposure route [37].

Evaluating human BPA intake via handling of thermal paper requires knowledge about the concentrations of BPA in these materials, the time that materials are held, the surface area of skin in contact with the papers, the rate of transfer from the paper to the skin surface, the number of handling events experienced daily, and the percentage of transferred compound that is absorbed through the skin into the bloodstream. Several of these factors have been measured experimentally. Yet, the behavioral aspects of this issue (e.g., how people actually interact with thermal paper) have been largely unstudied, and thus assumptions have been used about these factors when estimating chemical intakes. For example, the European Food Safety Authority’s (EFSA) exposure model estimates one handling event per day for adolescents and adults as *typical* exposures, and 4.6 handling events per day as *high* exposures; each exposure was anticipated to last 10 seconds [38]. EFSA also estimates that *average* exposure involves handling by three fingertips on one hand, and *high* exposure comprises handling by three fingertips on both hands (6 fingers total). To our knowledge, these behavioral attributes have not been evaluated experimentally.

Here, we have observed patterns of thermal paper handling in a college-aged population purchasing short-order food in a cafeteria environment. Almost 700 individuals were observed and data were collected for the length of time they handled the receipt and the surface areas (number of fingertips, palm of hand) in contact with the thermal paper. We paired these results with published values for other factors including the concentrations of BPA in thermal receipt papers (c), the rate of transfer from the paper to the skin surface (k), and the percentage of transferred compound that is absorbed through the skin into the bloodstream (AF) to calculate the anticipated intake from a single handling event.

## Materials and methods

### Ethical approvals

The experiments described in this manuscript were reviewed and approved by the University of Massachusetts Institutional Review Board (IRB). Although the UMass IRB determined that this study was “exempt” because it is purely observational, a protocol was placed on file for this study (number 2015–2726). Individuals observed in this study did not provide consent (written or verbal); the need for informed consent for this observational study was waived by the UMass IRB.

### Observations

#### Observational Selection Criteria—Location

During each observational session, an arbitrarily selected section of the Blue Wall dining concourse on the University of Massachusetts Amherst Campus was observed. The same area of the dining concourse was not observed on two consecutive days. During each observational session, individuals from a single dining area were examined. Dining areas included stations for Thai/Chinese food, Tacos, Salad, Pizza/Pasta, Grilled food, Sushi/Japanese, Deli Sandwiches, and “Chef’s Table” (a hot meal that changes daily).

#### Participant Inclusion/Exclusion Criteria

Each participant observed was estimated to be college aged based solely on researcher discretion. They were required to obtain a receipt from the dining staff. If they did not accept a receipt they were not included in this study. Participants were observed from the time they received the receipt until they stopped handling the receipt entirely; individuals that kept the receipt during the consumption of their meal were watched until the receipt was discarded or they left the dining concourse. Participants that did not stay within the direct visual field of the researcher were disqualified from the study. Researchers did not interact with participants.

#### Observational Selection Criteria—Participant

To select participants, the first observation was collected from the first individual that received their receipt during the observation period. The individual was observed continuously until they had discarded the receipt, put the receipt in a place that was no longer visible (e.g., a wallet or pocket), or left the dining concourse. Once the participant discarded the receipt, the observational period was over; individuals that temporarily ceased contact with the receipt (e.g. the receipt placed on the table during the consumption of their meal) were watched until the receipt was eventually discarded or the receipt was no longer visible (e.g., placed in a pocket). At the conclusion of this process, the next individual to receive a receipt at the dining area of interest was observed. This process continued until the allotted observation time had expired, typically a period of 2–4 hours.

#### Observational Data Collected

Data collected in this phase of experimentation included: date of interaction, assumed gender of participant, time in contact with receipt (from initial observed contact until the participant disposed of the receipt), maximum number of hand surfaces in contact with the receipt (number of fingers, palm), if and how many times the receipt was folded (maximum). In addition, a note of any abnormal behaviors was recorded.

Each individual that was observed (n = 698) was timed, using a standard stopwatch, from the time that individual first came in contact with the receipt until it was discarded or contact was otherwise stopped (e.g. placement in a wallet or pocket). Total handling time was rounded down to the nearest minute. For example, if the researcher observed a participant for 5 min and 57 sec, the recorded time for that observation was 5 minutes.

### Statistical analysis

SPSS Version 22 was used for all statistical analyses. Data presented in the results section represent mean ± SEM. An ANOVA test with Bonferroni posthoc correction for multiple comparisons was used to compare contact time by location within the dining facility.

#### Values used in modeling calculations

Our measurements of dermal handling (time, number of surfaces in contact with the paper) and Eq 1 were used to estimate intake from dermal BPA exposures. Other constants were determined or calculated from prior studies as described below.

The concentrations of BPA measured in thermal paper (c) reported in a number of studies range from 211 μg/g, the geometric mean of 103 thermal receipt papers reported by Liao and Kannan [24], to 13.3 mg/g, the mean reported by Biedermann et al. [23], to 26.3 mg/g, the maximum concentration reported by Hormann et al. [22]. Similarly, transfer coefficients (k, calculated as the amount transferred to skin divided by the product of the concentration in paper and the time handled) have been calculated as 21522 ng/s [23], 1072 ng/s or 1838 ng/s [22]. Finally, a wide range of absorption factors (AF) have been calculated from different experimental systems: 2.3–8.6% from skin explants [39], 27% from live hands after application of BPA [23] and 46–65% from human and pig skin explants [40]. These values were used as described in the results section to calculate estimates of daily intake.

$$\text{EI}=\left[\left({\text{k}*\text{c}*\text{HF}*\text{HT}*\text{AF}/10}^{6}\right)*\text{SF}\right]/\text{BW}$$ (1)

EI, Estimated intake

k, paper to skin transfer coefficient [ng / s]

c, concentration of BPA in thermal paper [μg / g paper]

HF, handling frequency, assigned a value of 1

HT, handling time, measured via observations

AF, absorption factor

SF, scaling factor for surface area, measured via observations

BW, body weight, assigned a value of 70 kg

## Results

### Typical handling of thermal paper in short-order cafeterias

The University of Massachusetts–Amherst has a central dining facility with multiple food stations where food is produced on short order; customers in this facility typically order their food, receive a receipt, and then wait for their food to be prepared, allowing for observations of typical thermal paper handling. Observational data were collected from 698 college aged individuals purchasing food in this dining facility. Females accounted for 364 of the observed participants (52%).

**Fig 1:**
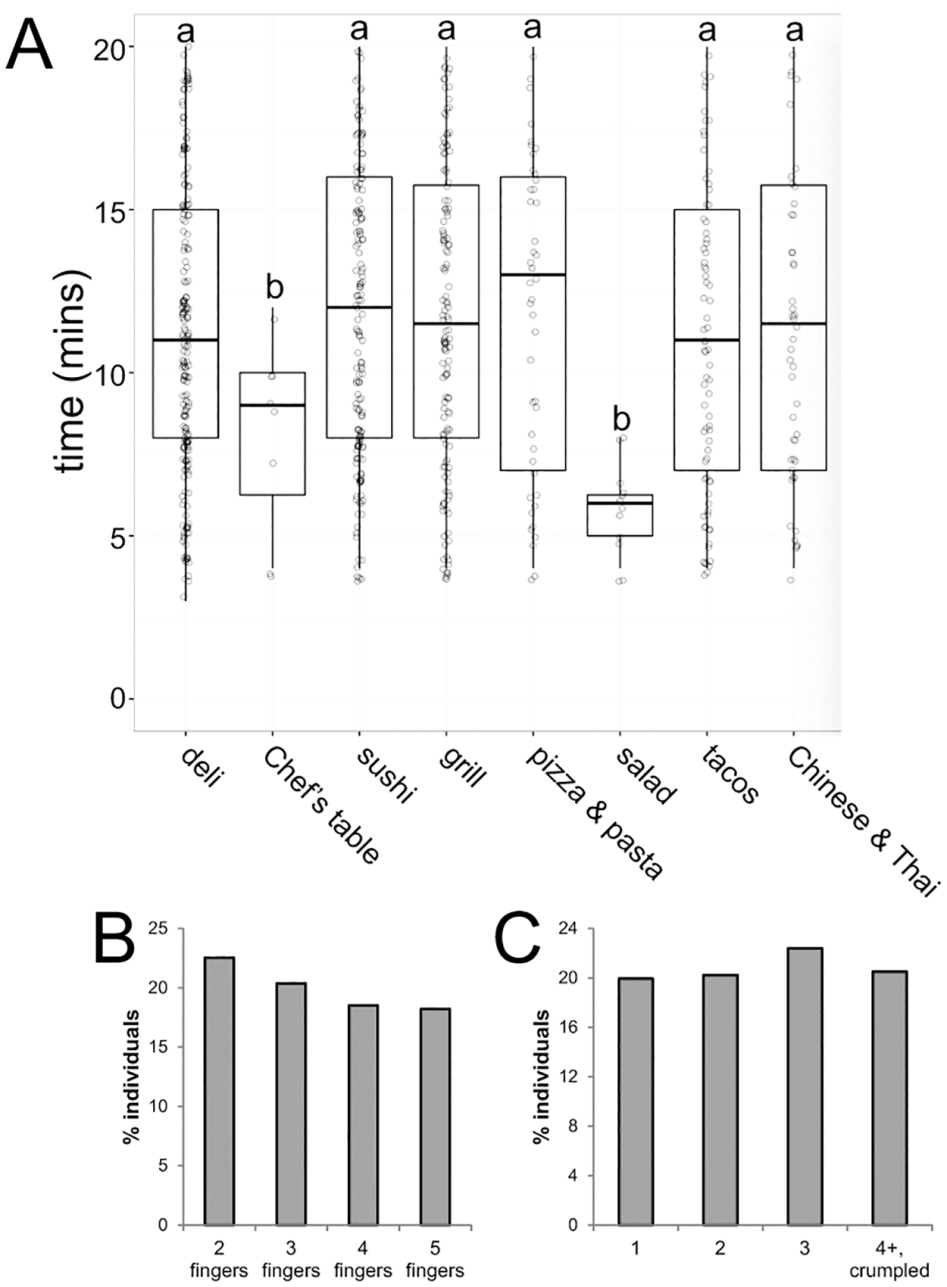
Characteristics of thermal paper handling by study participants. A) In most of the dining stations, individuals handled thermal paper for 10–12 minutes. Two stations were associated with decreased handling times, the salad station and Chef’s Table. Both of these stations involve preparing food prior to the acceptance of the receipt. Groups with different superscripts (letters a, b) indicate significant differences, p<0.05, Bonferroni adjusted posthoc comparisons after significant 1-way ANOVA. B) Characteristics of the number of fingers individuals used to handle thermal receipts. C) Characteristics of the number of folds participants made when handling thermal receipts.

Of the individuals observed, an average total contact time of 11.5 ± 0.26 min was measured. Similar average contact times were observed for females (11.6 ± 0.24 min) and males (11.3 ± 0.26 min). Some statistically significant differences were observed based on the type of food ordered; stations where food was prepared prior to the production of the receipt (e.g. the salad station) were associated with shorter handling times whereas stations where participants were typically handed a receipt followed by a wait for the food to be prepared (e.g. the sandwich station) were associated with significantly longer handling times (Fig 1A).

Observational data revealed a similar fraction of individuals that held the paper with two, three, four or five fingers (Fig 1B). Additionally, 63% of the participants were observed holding the receipt in their palm at some point during the observation. Finally, 83% of individuals folded their receipts, including 21% that folded their receipt four or more times or crumpled their receipt (Fig 1C).

### Abnormal handling patterns were observed

**Fig 2:**
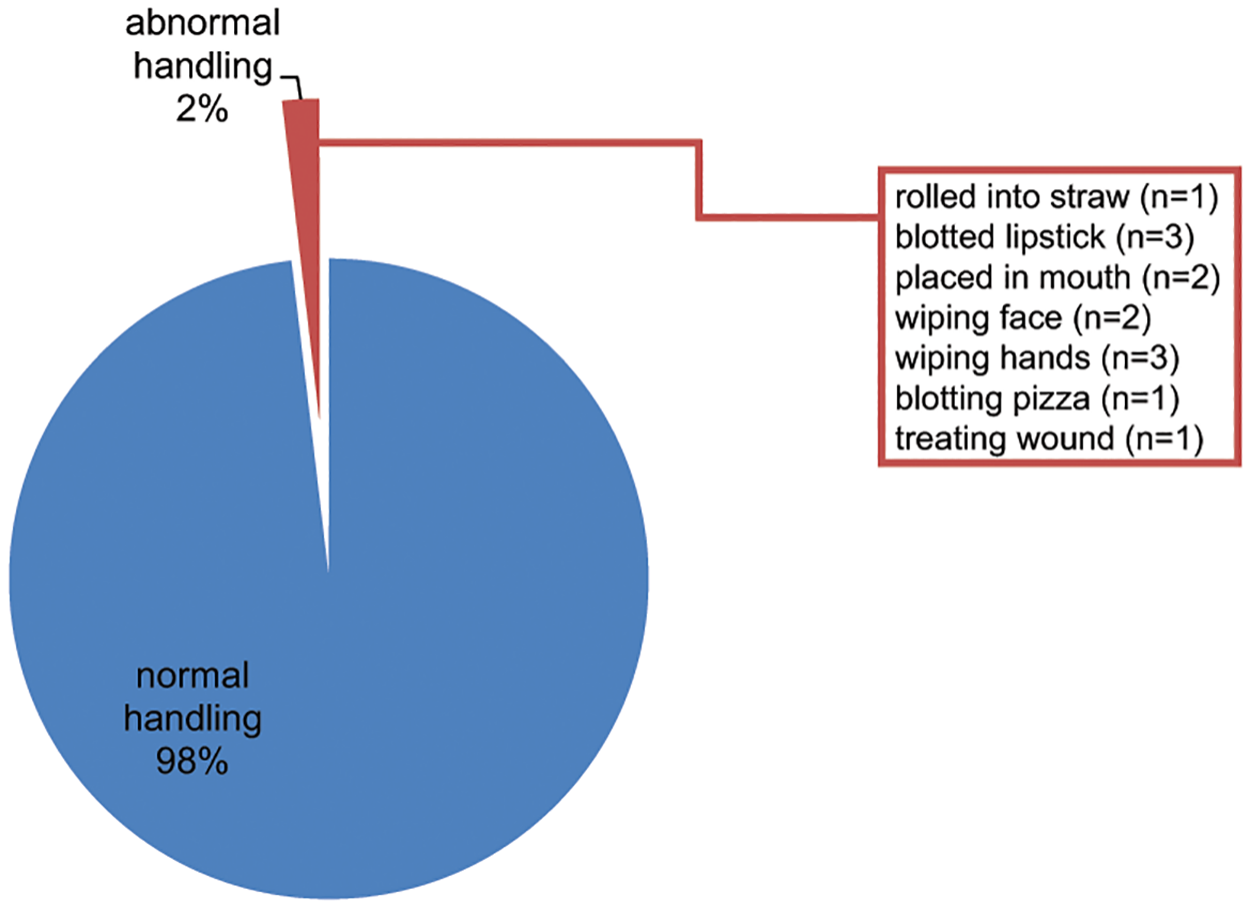
Abnormal handling parameters were observed in ~2% of study participants. Typical handling of thermal paper involves contact of only the hands (fingertips and palm) with the paper. Atypical handling patterns were observed in 13 individuals as indicated in the figure.

Participants were also evaluated for interactions with thermal paper in an unanticipated way. Individuals were considered to have abnormal handling patterns if they handled the thermal paper in a way that deviates significantly from the intended use of the paper, which could potentially alter their exposure to chemicals in thermal paper. We observed 13 individuals (2%) interacting with their receipt in a manner that was not anticipated from typical usage patterns (Fig 2). One subject was observed taking the receipt, rolling it into a cylinder and using it as a drinking straw. Three individuals used the receipt to blot lipstick and two additional participants placed their receipt in their mouth while handling other objects (plates, wallets, cell phones, etc.). Two participants were seen using their receipts as napkins to remove food from their faces, and another three subjects were observed using the receipt to remove food from their hands. One individual used a receipt to blot the grease from his pizza and finally one individual used a receipt to stop bleeding from a small cut on his hand.

### Estimates of BPA intake from thermal paper handling: Influence of handling time

**Fig 3:**
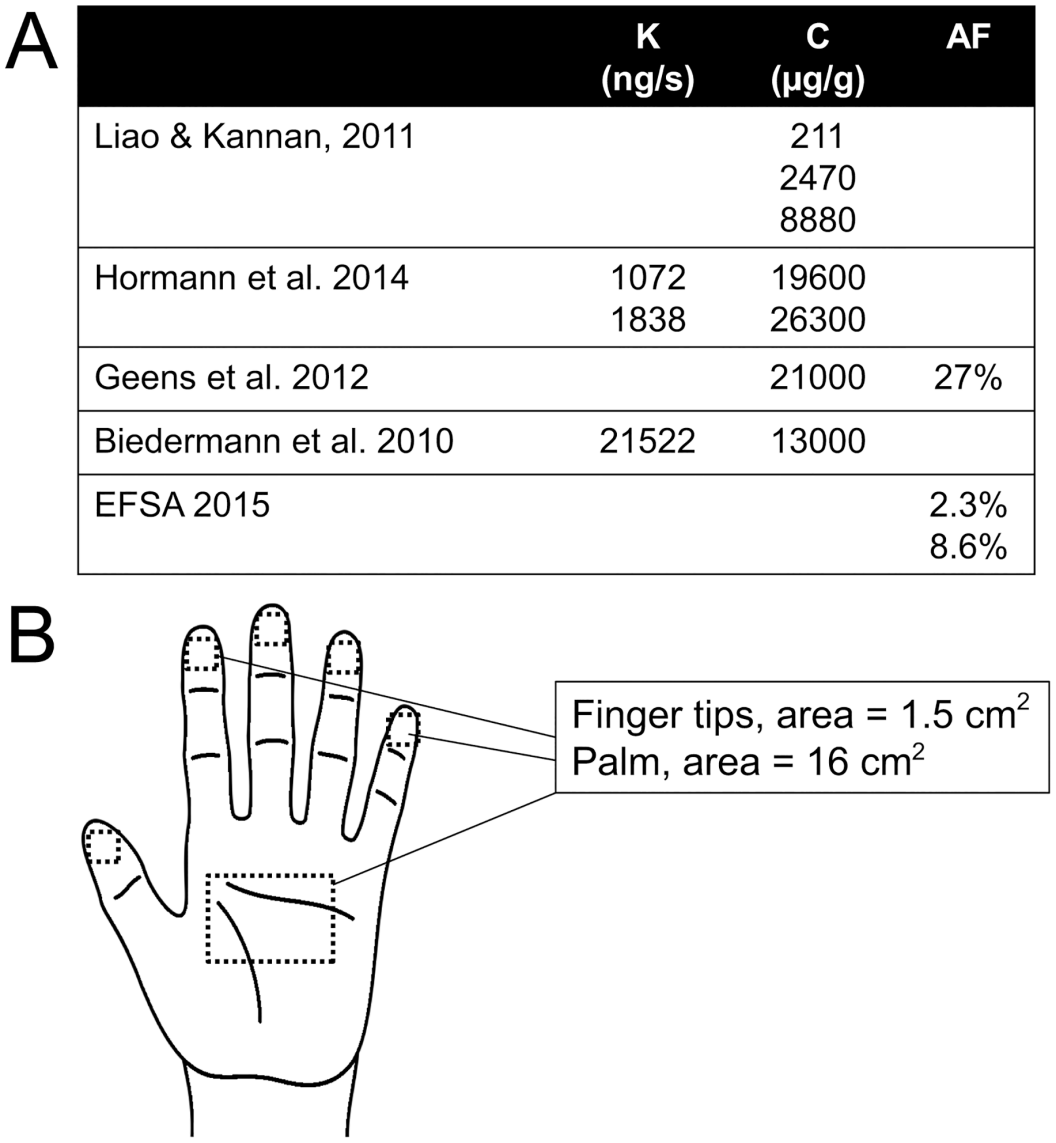
Factors and features that influence estimated intake. A) Summaries of transfer coefficients (k), concentrations (c) and absorption factors (AF) reported in prior studies. B) Conservative estimates of the surface area in contact with thermal paper held by fingertips or in the palm.

Liao and Kannan [24] used an equation to estimate BPA intake from thermal paper, with a number of constants and other variables that have been calculated or measured experimentally (Eq 1). Additional studies have allowed for new estimates for some of these variables (Fig 3A). To calculate plausible estimates of intake from thermal paper, we first calculated estimated intakes using the equation described in Eq 1, three different values of paper to skin transfer coefficients calculated from previous studies (k = 1072, 1838 or 21522 ng/s), three different values of absorption factors (AF = 2.3, 8.6 or 27%), and handling times measured in our observational study. Calculations were run for the lowest concentrations reported in thermal paper (0.211 mg/g) or the highest concentrations reported in thermal paper (26.3 mg/g).

Using the lowest concentrations reported in thermal paper, the most conservative estimate of intake is 51.1 ± 0.80 ng/kg/day; using the highest concentrations reported in thermal paper, the most conservative estimate of intake is 6298 ± 98.8 ng/kg/day (Tables 1 and 2). Calculations using the highest skin transfer coefficients and highest absorption factors suggest much higher intake levels (Tables 1 and 2).

**Table 1:** Effect of handling time on estimated intake when concentrations in thermal paper are low.

	K = 1072	K = 1838	K = 21522
**AF = 2.3%**	51.1 ± 0.80	87.6 ± 1.37	1026.2 ± 16.09
**AF = 8.6%**	191.1 ± 3.00	327.7 ± 5.14	3836.9 ± 60.18
**AF = 27%**	600.0 ± 9.41	1028.8 ± 16.14	12046.1 ± 188.94

Estimated exposure calculated using Eq 1, a single handling event, and thermal paper concentrations of 211 μg/g, the geometric mean reported previously by Liao & Kannan [25]. Scaling factors to account for different handling characteristics were not considered (see Eq 1).

**Table 2:** Effect of handling time on estimated intake when concentrations in thermal paper are high.

	K = 1072	K = 1838	K = 21522
**AF = 2.3%**	6298 ± 98.8	10799 ± 169.4	126445 ± 1983.2
**AF = 8.6%**	23550 ± 369.4	40377 ± 633.3	472793 ± 7415.5
**AF = 27%**	73935 ± 1159.6	126765 ± 1988.2	1484351 ± 23281.3

Estimated exposure calculated using Eq 1, a single handling event, and thermal paper concentrations of 26.3 mg/g, the maximum reported by Hormann et al. [22]. Scaling factors to account for different handling characteristics were not considered (see Eq 1).

### Estimates of BPA intake from thermal paper handling: Influence of surface area in contact with thermal paper

Studies by Biedermann and colleagues [23] have suggested that handling time is less important than surface area in contact with thermal paper, leading us to add a scaling factor to estimate surface area in contact with paper based on handling characteristics (Eq 1, Fig 3B).

Using Eq 1, a maximum handling time of 240 seconds, and scaling factors to account for different handling characteristics, we again calculated intake using three different values of skin transfer coefficients and three different absorption factors. When calculations evaluated intake using the lowest concentrations reported in thermal paper (0.211 mg/g), the most conservative estimate of intake is 85.9 ± 1.81 ng/kg/day (Table 3). Calculations using the highest concentrations reported in thermal paper (26.3 mg/g) produce conservative estimates of 10582.9 ± 223.3 ng/kg/day (Table 4). Calculations using the highest skin transfer coefficients and highest absorption factors again suggest much higher intake levels (Tables 3 and 4).

**Table 3:** Effect of handling surface area on estimated intake when concentrations in thermal paper are low.

	K = 1072	K = 1838	K = 21522
**AF = 2.3%**	85.9 ± 1.81	147.3 ± 3.11	1724.3 ± 36.4
**AF = 8.6%**	321.1 ± 6.78	550.6 ± 11.62	6447.2 ± 136.0
**AF = 27%**	1008.2 ± 21.3	1728.6 ± 36.47	20241.2 ± 427.1

Estimated exposure calculated using Eq 1, a single handling event, maximum handling time of 240 seconds and thermal paper concentrations of 211 μg/g, the geometric mean reported previously by Liao & Kannan [25]. Surface area was calculated using the values described in Fig 3B.

**Table 4:** Effect of handling surface area on estimated intake when concentrations in thermal paper are high.

	K = 1072	K = 1838	K = 21522
**AF = 2.3%**	10582.9 ± 223.3	18145 ± 382.8	212467 ± 4483
**AF = 8.6%**	39571 ± 834.9	67846 ± 1432	794441 ± 16762
**AF = 27%**	124234 ± 2621	213005 ± 4494	2494175 ± 52626

Estimated exposure calculated using Eq 1, a single handling event, maximum handling time of 240 seconds and thermal paper concentrations of 26.3 mg/g, the maximum reported by Hormann et al. [22]. Surface area was calculated using the values described in Fig 3B.

## Discussion

In this study, we provide observational data describing the way that individuals interact with thermal paper in a short-order cafeteria setting. In this setting, individuals typically placed an order for food, received a receipt, and then held that receipt (with their order number) until their food was prepared. The average individual held the thermal paper for more than 11 minutes with multiple fingers and the palm of their hand (Fig 1). These average interactions deviate significantly from the assumptions that EFSA included in their estimates of exposures from the handling of thermal papers. In fact, only 11% of the participants we observed matched the EFSA model for time of contact and dermal surface area. Furthermore, approximately 2% of the interactions we observed involved atypical handling practices (Fig 2) that might be anticipated to significantly increase BPA intake, and may also involve intake via other exposure routes (e.g., oral via contact with the mouth or ingestion via food). In 2015, EFSA wrote that “children chewing paper receipts is assumed to occur only sporadically, so that no chronic exposure results” [38]. Our observations suggest that assumptions about ‘atypical’ interactions with thermal paper may need to be revisited, both for children and adults.

Because observational data on handling of thermal paper was lacking prior to this study, we specifically focused on two important variables, handling time and surface area in contact with thermal paper. Intake from dermal exposure is influenced by a number of additional factors including the concentration of BPA in the thermal paper (c), the transfer coefficient (k), the number of handling events per day (HF), the absorption factor (AF), and body weight (BW) (Eq 1, Fig 3A). A number of studies have evaluated the concentrations of BPA in thermal paper, and these report a wide range of values: as low as 211 μg/g, the geometric mean of 103 thermal receipt papers [24], to 13.3 mg/g, the mean of eleven thermal paper samples containing BPA [23], to 26.3 mg/g, the maximum concentration reported in an evaluation of 50 thermal papers [22]. Studies describing the chemistry of thermal paper manufacturing suggest that milligrams of developer are needed per gram of paper [21]. This suggests that the very low concentrations reported by Liao and Kannan [24] might be because these thermal papers were using a different chemical developer (e.g., bisphenol S) [41]. The low level of BPA measured in these papers could be due to a contamination or transfer from papers containing a BPA developer. If true, the values reported in Tables 1 and 3 would be a gross underestimation of exposures from the handling of typical thermal papers; the intake levels reported in Tables 2 and 4 are therefore likely better estimates of exposure from handling of typical thermal papers.

Several different transfer coefficients (calculated as the amount transferred to skin divided by the product of the concentration in paper and the time handled) have also been calculated using data provided in different studies: 21522 ng/s [23], 1072 ng/s or 1838 ng/s [22]. At this time, it is difficult to determine how manufacturing practices and other features of the thermal paper might affect transfer from paper to skin. Furthermore, although BPA does not appear to re-transfer from hands to dry material [42], it likely can be transferred from hands to wet or oily foods [22], contributing to oral exposures. We examined receipts from a small number of participants and found that these receipts often had grease spots on them, suggesting that there may have been direct contact between the thermal paper and foods (S1 Fig). These are important issues that should be evaluated in updated toxicokinetic models evaluating exposures.

A related issue raised by Hormann and colleagues is the use of personal care products containing dermal penetrants such as hand sanitizers and some soaps and lotions [22]. More than 300 different chemicals have been identified as permeation enhancers, and these are used in drug delivery systems including gels, patches, and topical creams as well as non-pharmaceutical personal care products [43]. Hormann et al. showed that the use of hand sanitizer just prior to the handling of receipts increased transfer to the skin by factors of 22–200 times [22]. In fact, calculations of transfer coefficients from studies of individuals with wet hands revealed k-values ranging from 474673 ng/s for longer handling times (45 s) to 4,319,853 ng/s for shorter handling times (2 s).

For many years, it was assumed that the skin was impermeable to “any substance”, but since World War II, when dermal treatments for a number of conditions including insect repellents and prophylactics for venereal diseases were developed, it has been understood that the skin can act as a “reservoir” for both drugs and non-pharmaceutical chemicals [44]. Mathematical modeling using the physiochemical properties of BPA suggest that it will be readily absorbed by intact human skin [45]. In fact, a wide range of absorption factors have been calculated in different experimental systems. Using the in vitro OECD guideline skin test, Demierre and colleagues measured absorption of 2.3–8.6% of BPA applied to skin explants [39]. In contrast, Biedermann et al. calculated absorption of 27% based on swabs taken from live hands after application of BPA to skin [23]. Finally, Zalko and colleagues used pig and human skin explants to estimate absorption factors of 46–65% [40]. The variability in these three factors contributes significantly to the wide range of estimates of intake from dermal contact (Tables 1–4).

Finally, an important factor in calculating intake is the body weight of the exposed individual. Here, as in the EFSA model, we used 70 kg as an estimate of ‘average’ body weight. However, it should be noted that individuals that weigh less than 70 kg will have higher intakes relative to individuals with the ‘average’ body weight, and individuals that weigh more than 70 kg will have lower relative intakes. Body weight information was not collected, or estimated, for the subjects observed in this study.

We selected a college-aged population for this study due to its convenience and the vulnerability of the population, which is sexually developed but still in the age range where brain development is occurring [46, 47]. More importantly, we were interested in thermal paper handling in this specific type of setting; here, individuals were provided a receipt with an order number on it, similar to how people may interact with thermal paper in other cafeterias, fast food establishments, or lines in grocery markets or train stations and airports. Our results are likely not generalizable to all interactions with thermal paper; handling of thermal paper after shorter transactions (e.g., purchases at the grocery store, gas stations) or visits to the ATM are expected to be shorter in duration, although the number of surfaces (fingers, palm) in contact with the paper may be similar. Additional studies are needed to evaluate handling practices in these other settings.

This study did not evaluate the concentrations of BPA in thermal receipt papers, or even determine that the thermal paper being used at the University of Massachusetts dining facility contained BPA rather than one of the alternative chemicals that have been measured in this product [41]. There is no reason to believe that individuals would handle thermal paper differently if it contains BPA or another compound. However, estimated intakes of these alternative compounds would depend on their concentration in thermal paper, transfer coefficients, and their absorption factors, which could be influenced by physiochemical properties of the compound. Bisphenol S (BPS) is a common replacement for BPA in thermal paper [22, 25, 41, 48]. Although it is not yet well studied, there is now some evidence that BPS can also affect estrogen-sensitive endpoints in exposed animals [49–52]. Human exposures to BPS are low but widespread [48] and have increased over the last decade [53], suggesting that this and other BPA replacements deserve additional attention [54].

## Conclusions

Only in the last few years have regulatory agencies and risk assessors acknowledged that human exposure to BPA occurs via non-oral and non-dietary sources. The EFSA model estimates typically daily dermal exposures of 59 ng/kg body weight [38]. These levels are consistent with our best-case scenario (extremely low concentration in thermal paper [perhaps because a different developer was used], lowest transfer coefficient, and lowest absorption factor), which predicts dermal intake levels of 51.1 ng/kg body weight after a single handling event. If any of the factors that are used to calculate intake are underestimates–and there is experimental evidence to suggest that they are–actual exposures experienced by populations like the ones we observed here are likely to be much higher. Our study reveals the importance of conducting observational studies to evaluate actual consumer behaviors rather than relying on assumptions that underestimate usage patterns.

## Supporting information

S1 Fig Image showing typical receipt, with area containing grease outlined. (TIF)

S1 File Data collected underlying the analysis presented in this manuscript. Coded data. Gender: 1 = female, 2 = male; Location: 1 = deli, 2 = Chef’s table, 3 = sushi, 4 = grill, 5 = pizza & pasta, 6 = salad, 7 = taco, 8 = Chinese & Thai.
(XLSX)
