# Supplementary figures and images for: Handling of thermal paper: Implications for dermal exposure to bisphenol A and its alternatives
Source: PLoS One. 2017 Jun 1;12(6):e0178449. doi: 10.1371/journal.pone.0178449 (PMC5453537; doi:10.1371/journal.pone.0178449)

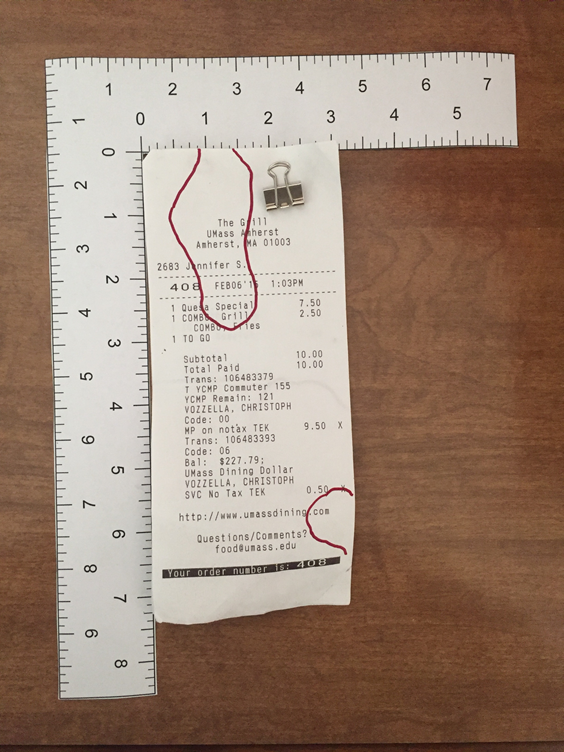

Supplement: S1 Fig — (TIF) [file pone.0178449.s001.tif]
